# Anti-inflammatory Effects and Mechanisms of Rhein, an Anthraquinone Compound, and Its Applications in Treating Arthritis: A Review

**DOI:** 10.1007/s13659-020-00272-y

**Published:** 2020-10-30

**Authors:** Hongjuan Wang, Dezhi Yang, Li Li, Shiying Yang, Guanhua Du, Yang Lu

**Affiliations:** 1 Beijing Key Laboratory of Polymorphic Drugs, Center of Pharmaceutical Polymorphs, Institute of Materia Medica, Chinese Academy of Medical Sciences and Peking Union Medical College, Beijing, 100050 China; 2 Beijing Key Laboratory of Drug Targets Identification and Drug Screening, National Center for Pharmaceutical Screening, Institute of Materia Medica, Chinese Academy of Medical Sciences and Peking Union Medical College, Beijing, 100050 China

**Keywords:** Rhein, Anthraquinone, Anti-inflammatory, Arthritis

## Abstract

Inflammation is a defensive response of living tissues to damaging agents, which exists in two forms, acute inflammation and chronic inflammation, and chronic inflammation is closely related to arthritis. Currently, the commonly prescribed anti-inflammatory medications are greatly limited by high incidence of gastrointestinal erosions in the clinical applications. Rhein, a bioactive constituent of anthraquinone, exhibits excellent anti-inflammatory activities and therapeutic effects on arthritis with less gastrointestinal damages. Although there are numbers of studies on anti-inflammatory effects and mechanisms of rhein in the last few decades, to the best of our knowledge, only a few review articles pay attention to the interactive relationships of rhein on multiple inflammatory signaling pathways and cellular processes from a comprehensive perspective. Herein, we summarized anti-inflammatory effects and mechanisms of rhein and its practical applications in the treatment of arthritis, thereby providing a reference for its basic researches and clinical applications.

## Introduction

Inflammation is a defense response process generated by the body [1], the essence of which is homeostasis imbalance between pro-inflammatory and anti-inflammatory [2]. Inflammation exists in two forms, acute inflammation and chronic inflammation, and chronic inflammatory responses are closely related to various arthritis [3–5] including rheumatoid arthritis (RA) [6–8], osteoarthritis (OA) [9–11], gouty arthritis (GA) [12–15] and reactive arthritis [16]. In general, there are numbers of lymphocytes, macrophages and other inflammatory cells in joint cavity, which release inflammatory mediators and then activate the body's autoimmunity, so it is the key point to block the secretion of inflammatory cells and inflammatory mediators for the treatment of arthritis. Up to now, the commonly prescribed anti-inflammatory agents mainly include steroidal anti-inflammatory drugs (SAIDs), non-steroidal anti-inflammatory drugs (NSAIDs) and cyclooxygenase inhibitors [17–19]. Despite suppressing inflammatory features and so improving the quality of life for arthritis patients, steroids and cyclooxygenase inhibitors are symptom-suppressing drugs by easing pain and diminishing swelling in treating arthritis [20, 21]. What's worse, NSAIDs may lead to serious side effects including peptic ulceration [22, 23], gastrointestinal damage [24] and ulcer complications [25].

Medicinal plants play a vital role as sources of active anti-inflammatory ingredients in the prevention of various inflammatory diseases. Rhein, a bioactive constituent of anthraquinone, has been well recognized for its excellent anti-inflammatory activities and therapeutic effects on arthritis [26]. A growing body of researches have demonstrated that rhein exhibits anti-inflammatory activities by inhibiting cytokines [27, 28] and interleukins [29] which cause inflammatory responses and metabolic abnormalities, stimulates the formation of cartilage matrix material and promotes cartilage repair to remodel joint structures [30, 31]. Nguyen et al. [32] investigated that the effects of rhein on osteoarthritis (OA) and found it inhibits the production and release of inflammatory factors. Pelletier et al. [33] found that rhein could inhibit the production of NO and block the expression of inducible NO synthase. Legendre et al. [34] found that rhein could restrain the release of the inflammatory mediators, inhibit arthritic lymphocyte chemotaxis and promote cell apoptosis in type II collagen-induced rheumatoid arthritis (RA).

Although a growing number of researches focus on the anti-inflammatory effects and mechanisms of rhein for treating arthritis [35–40], to the best of our knowledge, the mechanisms underlying these beneficial effects are not completely investigated from a comprehensive perspective. More importantly, there are no review articles that pay attention to the interactive regulations of rhein on multiple inflammatory signaling pathways. For instance, rhein blocks the activation of nuclear transcription factor-kB (NF-κB) and sequentially suppresses interleukin-1β (IL-1β) transcription, but at the same time, it enhances the activity of caspase-1 by blocking inhibitor of nuclear factor -kB kinase β (IKKβ), which in turn increasing the production of IL-1β. Therefore, it can be speculated that the regulations of rhein on the inflammatory responses is the result of multiple signaling pathways interactions. This article reviewed the anti-inflammatory effects and mechanisms of rhein and its practical applications in treating arthritis, thereby providing reference for the basic researches and clinical applications.

## The Regulations of Rhein on Inflammatory Signaling Pathways and Cellular Processes

### Nuclear Transcription Factor-kB (NF-kB) Signaling Pathway

Gao et al. [41] found that rhein blocks the activation of NF-κB pathway and thus inhibits tumor necrosis factor-α (TNF-α) and interleukin-1β (IL-1β) transcription by inhibiting IKKβ in LPS-activated macrophages. But at the same time, it enhances the activity of caspase-1 by suppressing IKKβ, which in turn activating the production and release of IL-1β. Therefore, it can be summarized that rhein exhibits anti-inflammatory and pro-inflammatory effects by inhibiting the production of IKKβ. In fact, the clinical application processes of IKKβ inhibitors, including rhein, are very complicated since their activities vary in different tissues and inflammatory processes. Herein, the stage of inflammation should be considered before using IKKβ inhibitors to ensure effectiveness and efficacy of the treatment.

**Fig. 1:**
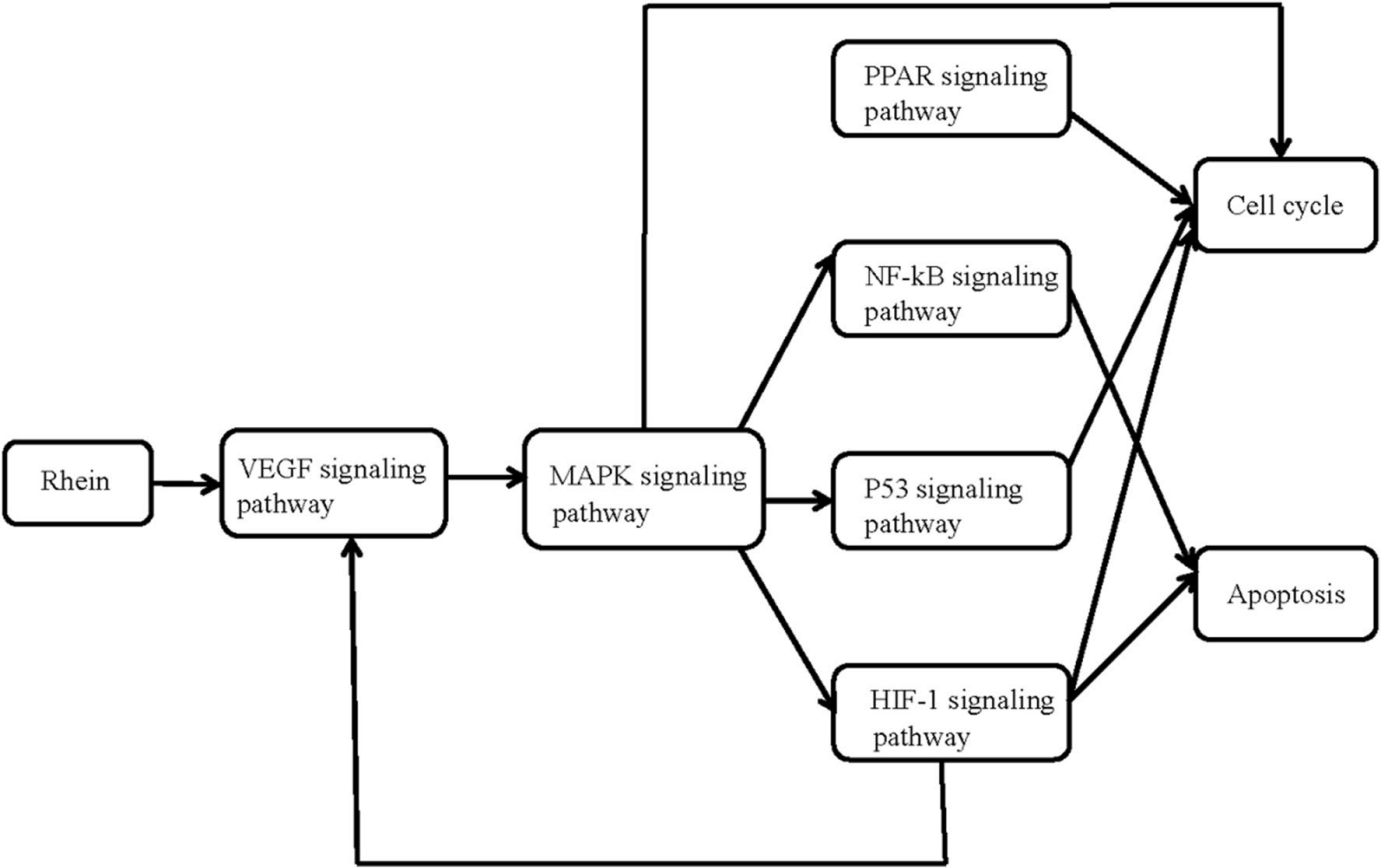
The anti-inflammatory effects and mechanisms signaling pathway diagram of rhein [27]

Mendes et al. [42] investigated the regulations of rhein on NF-kB transcription, induced by pro-inflammatory cytokine IL-1β, and also studied the ability of rhein to prevent the expression of the inducible nitric oxide (iNOS) gene, which is induced by NF-kB signaling pathway. The results suggested that rhein could block the degradation of IL-1β-induced inhibitor of nuclear factor kB-α (IkBα) and the translocation of protein p65, a member of the NF-kB family, to the nucleus in a dose-dependent manner. In addition, rhein could also suppress the synthesis of iNOS mRNA and protein and the production of NO. These results indicate that rhein could block the activation of NF-kB signaling pathway and thus the expression of NF-kB-related genes, demonstrating its anti-inflammatory effects in treating osteoarthritic (Fig. 1).

### Mitogen-Activated Protein Kinase (MAPK) Signaling Pathway

Mitogen-activated protein kinase (MAPK), a hub signaling molecule, serves as a key mediator of signaling transduction from cell surface to nucleus [43], which can be categorized as four main families, including extracellular regulated protein kinases (ERK), c-Jun amino-terminal kinase (JNK), p38 mitogen-activated protein kinase (p38 MAPK) and extracellular signaling-regulated kinase 5 (ERK5) [44]. Among them, JNK and p38 MAPK take primarily responsibilities for mediating inflammation and apoptosis. Lin et al. [45] found that rhein regulates multiple sites of MAPK signaling pathway by inhibiting the phosphorylation of ERK, JNK and p38 MAPK. It is worth mentioning that rhein inhibits the MAPK signaling pathway at a low dose. In fact, once inflammatory signaling transductions are inhibited, the proliferation and secretion of inflammatory mediators would be greatly reduced at the cellular level.

### Vascular Endothelial Growth Factor (VEGF) Signaling Pathway

Lee et al. [46] evaluated the part of VEGF in inflammation and found that it induces inflammation through IL-13-dependent signaling pathway. It is well-known that vascular endothelial growth factor receptor 2 (VEGFR-2), i.e. kinase insert domain receptor (KDR), is the key mediator of VEGF-driven responses in endothelial cells. Once VEGF binds to KDR, the downstream signaling pathways are perturbed which leads to the proliferation and migration of endothelial cells. He et al. [47] investigated that the suppression of rhein on VEGF pathway, and found that it could down-regulate the expression of vascular endothelial growth factor A (VEGFA) and receptor KDR, blocking angiogenesis and cell migration. Fernand et al. [48] found that rhein suppresses heat shock protein 90-α (Hsp90α), leading to the degradation of cyclooxygenase 2 (COX-2) and increasing the production and secretion of prostaglandin I2 (PGI2) which blocks the release of inflammatory mediators in the downstream of VEGF signaling pathway.

### Peroxisome Proliferators-Activated Receptors Pathway (PPARs)

It has been reported that cytokines including TNF-α have an inhibitory activity on the synthesis of PPARγ [49]. Antonisamy et al. [50] demonstrated that rhein has a significant effect on the expression of PPARγ, which is a ligand-activated transcription factors belonging to the nuclear hormone receptor family. They also found that rhein markedly increases the level of PPARγ in a dose-dependent manner, which can be explained by the fact that the inhibition of TNF-α mediated by rhein leads to the increasing of PPARγ, and consequently inhibits the release of inflammatory mediators such as iNOS and COX-2. Although different effects have been proposed for PPARγ during the onset of inflammation, it still remains unclear how inflammation is exactly affected by PPARγ pathway.

### The Regulations of Rhein on Cellular Processes

The P2X7 receptor plays a important role in inflammation which involved in the release of cytokines, and in particular, promotes the secretion of VEGF from monocytes, depending on ([Ca(^2+^)]c) and reactive oxygen species (ROS) production [51]. Hu et al. [52] investigated the regulations of rhein on inflammation induced by P2X7 receptor. The results showed that rhein, as a P2X7 channel blocker, inhibits ATP-induced cytosolic calcium concentration ([Ca(^2+^)]c) elevation and pore formation of the plasma membrane in a dose-dependent manner. Moreover, it offsets macrophage phagocytosis and suppresses the production of ROS. Therefore, it can be concluded that rhein inhibits the increasing of [Ca(^2+^)]c, the formation of pore, the production of ROS, phagocytosis attenuation and cell apoptosis by antagonizing P2X7 receptor in macrophages (Table 1).

**Table 1:** The regulations of rhein on inflammatory signaling pathways and cellular processes

Signaling pathway	Abbreviations	Action mechanisms	References
Nuclear transcription factor-kB signaling pathway	NF-kB	Rhein inhibits NF-κB activation and suppresses the transcription of downstream genes and inhibits NO and IL-6 levels by inhibiting IKKβ in LPS-activated macrophages. It also elevates the activity of Caspase-1 by inhibiting intracellular IKKβ, thereby increasing the release of IL-1β and HMGB1	[35]
		Rhein inhibits the degradation of the inhibitor of nuclear factor kB-α (IkBα) and regulates the translocation of the protein p65	[36]
Mitogen-activated protein kinase signaling pathway	MAPK	Rhein can regulate multiple sites of MAPK signaling pathways, and its targets are mainly involved in three signaling cascades, including ERK1/2, JNK, and p38 MAPK	[39]
Vascular endothelial growth factor signaling pathway	VEGF	Rhein can down regulate the expression of VEGFA and receptor KDR(VEGFR-2) to inhibit angiogenesis and cell migration	[41]
		In the downstream of VEGF signaling pathway, rhein inhibits Hsp90α activity to induce the degradation of its client protein COX-2 and to promote the production of PGI2	[42]
Peroxisome proliferators-activated receptors signaling pathway	PPARs	Rhein increases the level of PPARγ in a dose-dependent manner, which can be explained by the fact that the inhibition of TNF-α mediated by rhein leads to the increasing of PPARγ and, consequently, inhibits the release of inflammatory mediators	[43]
Cellular processes		Rhein inhibits ATP-induced cytosolic calcium concentration ([Ca(2 +)]c) elevation and pore formation of the plasma membrane in a dose-dependent manner. It also offsets macrophage phagocytosis and suppresses the production of ROS	[44]

## The Applications of Rhein in the Treatment of Arthritis

### The Effects of Rhein on Osteoarthritis (OA)

Osteoarthritis (OA) [53] is a degenerative disease marked by degenerative lesions of articular cartilage [54, 55]. It is reported that IL-1 [56] plays a significant part in the pathogenesis of OA by suppressing the synthesis of cartilage matrix components and promoting the degradation of cartilage matrix. Moreover, the production of NO [57] is the result of the cartilage catabolic process participating in OA pathogenesis.

Yaron et al. [58] assessed the effect of rhein on IL-1β, NO and interleukin-1 receptor agonist (IL-1ra) in osteoarthritic synovial and articular cartilage. The results showed that rhein has an inhibitory on IL-1β and IL-1ra in the synovial tissue while it suppresses IL-1β but enhances the production of IL-1ra in cartilage culture. In addition, it inhibits the production of NO in LPS-induced synovial tissue and cartilage cultures. Moldovan et al. [31] demonstrated rhein blocks the expression and production of interleukin-1β converting enzyme (ICE), and consequently, reduces the level of IL-1β in OA cartilage chondrocytes. Christelle Scanchez et al. believed that rhein depresses matrix metalloproteinase 3(MMP-3) synthesis and increases the production of tissue metalloproteinase inhibitor-1 (TIMP-1) since it has a positive effect on proteoglycan degradation and the activities of pro-MMPs, leading to the decrease of MMP-3/TIMP-1 ratio. In addition, rhein could increase the production of 1L-1β-stimulated prostaglandin E2 (PGE2). Actually, the effect of rhein on the synthesis of PGE2 can be explained by the fact that it has an inhibitory effect on NO and NO could down regulate the synthesis of PEG2.

### The Effects of Rhein on Rheumatoid Arthritis (RA)

Rheumatoid arthritis [59] (RA) is a chronic autoimmune disease with joint pathology, including chronic inflammation of the synovial membrane, inflammatory cell infiltration, the formation of vascular opacities and the destruction of cartilage tissues, resulting in joint deformity and loss of function [7, 60].

Fen et al. [61] determined the regulations of rhein on inflammation induced by ATP such as intracellular calcium [Ca^2+^] mobilization, ROS production and inflammatory gene expressions. The results indicated that rhein could dose-dependently inhibit the increasing of ATP-induced [Ca^2+^] and suppress the production of intracellular ROS. Furthermore, they demonstrated that P2X4 receptors are responsible for the increase of ATP-induced [Ca^2+^], ROS production, as well as inflammatory gene expression, demonstrating that the regulations of rhein on ATP-induced inflammatory responses contribute to the antagonism of P2X4. Martin et al. [62] found that the severity of RA is associated with the cytokines IL-1β and TNF-α, and rhein regulates RA by inhibiting the expression and activity of pro-inflammatory cytokines TNF-α, IL-6, IL-8 and PGE2. Zippel et al. [63] found that rhein inhibits the over expression of inflammatory factors by reducing VEGF and hippocampal tissue hypoxia-inducible factor-1α (HIF-1α) in vascular synovial fluid, thereby blocking the proliferation of RA synovial cells. LO Demirezer et al. [64] found that matrix metalloproteinase (MMP) released by synovial cells is associated with joint destruction in rheumatoid pathogenesis and that rhein inhibits the up regulation of MMP-1 and MMP-13 expression in synovial cells.

### The Effects of Rhein on Gouty Arthritis (GA)

Gouty arthritis is marked by hyperuricemia and inflammation induced by urate crystal [65]. The deposition of urate crystals in joints contributes to aggregation of phagocytes and leads to innate immune response, leading to the formation of intracellular NACHT, LRR and PYD domains-containing protein 3 (NLRP3) inflammasome [66]. Therefore, the activation of NLRP3 and the secretion of IL-1β are regarded as the key step in the onset of inflammation of GA, and suppressing the formation of NLRP3 has been viewed as a potential therapeutic target for the treatment and prevention of GA [67, 68].

Chang et al. [26] evaluated the anti-inflammatory activities of rhein on GA and found that it significantly decreases IL-1β production induced by urate crystal by interrupting NLRP3 formation and suppressing caspase 1 (CASP1) protease activity within the physiological levels. In addition, rhein inhibits the transport activity of the renal organic anion transporter (OAT), resulting in the reduction of uric acid re-uptake. Meng et al. [40] investigated the regulations of rhein on hyperuricemia and found that rhein greatly down regulates the serum uric acid level by inhibiting the xanthine oxidase (XOD) activity and enhancing the excretion of urinary uric acid. Further, it is well recognized that hyperuricemia could be reduced by blocking XOD activity or promoting excretion of uric acid. They also found that rhein could suppress the activity of XOD and enhance the excretion of uric acid.

It can be concluded that rhein alleviates the symptom of uric acid at least through two pathways, namely, increasing uric acid excretion and reducing the inflammatory responses, possibly owning to the down-regulation of IL-1β, TNF-α and PGE2. Furthermore, it is the effects of interrupting NLRP3 formation and blocking OAT activity that may make rhein possess the possibility for its clinical application in treating GA and other NLRP3 inflammasome-associated diseases.

### The Effects of Rhein on Other Osteoarthritic Conditions

The physiological state of the bone tissue is in a state of constant renewal, accomplished by both osteogenic and osteoclastic cells in concert [69]. Under normal conditions, the two cells are in a dynamic equilibrium to maintain the normal functioning of bone tissue; under pathological conditions, the balance is disrupted and diseases dominated by bone resorption, such as osteoporosis and other arthropathies appear [3]. The formation of mature osteoclasts is related to tartaric acid resistant acid phosphate (TRAP), calcitonin receptor (CTR), and carbonic anhydrase II (CAII).

Xu et al. [70] explored the regulations of rhein on the formation and differentiation of osteoblasts, and found that rhein mainly affects the mid-late stage apoptosis of cells through the apoptotic pathway. Pelletier et al. [71] investigated the effects of rhein on osteoclast bone resorption activity, and found that it could inhibit bone resorption in osteoclast, which achieved by inhibiting the activities of TRAP and tissue proteinase K. In addition, it also enhances macrophage colony-stimulating factor (M-CSF)-induced bone marrow osteoclast apoptosis and decreases CTR expression. Therefore, it can be concluded that osteoclast bone resorption is mediated by osteoclast formation and osteoclast resorption activity, and rhein reduces bone resorption and osteoclast resorption plaque primarily by inhibiting the formation of muti-nucleated osteoclasts. The treatment of bone resorption diseases with increased osteoclast activity as the main focus of rhein is expected to be a novel therapeutic approach for arthritis in the near future (Table 2).

**Table 2:** The anti-inflammatory effects and mechanisms of rhein on various arthritis

Types of arthritis	Characteristics	Mechanisms	References
Osteoarthritis (OA)	Degenerative lesions of articular cartilage	Rhein inhibits LPS-induced IL-1β production by synovial tissue and cartilage, and reverses the inhibitory effect of LPS on cartilage^35^S uptake	[58]
		Rhein suppresses the interleukin-1b converting enzyme(ICE), blocks matrix metalloproteinase 3(MMP-3) synthesis, and increases the production of prostaglandin E2 (PGE2)	[7]
Rheumatoid arthritis (RA)	Inflammatory synovitis and progressive joint lesions	Rhein could block the ATP-induced [Ca^2+^]c increases in a dose-dependent manner. Besides, rhein could also suppress the production of intracellular reactive oxygen species (ROS) induced by ATP in synoviocytes that was resulted from P2X4-mediated Ca^2+^ entry	[61]
		Rhein can effectively inhibit the IL-1-activated MAPK pathway and the binding of NF-κB and AP-1 transcription factors. In addition, rhein can reduce the procatabolic effect of the cytokine, by reducing the MMP1 synthesis, and enhance the synthesis of matrix components	[62]
		Rhein reduces VEGF and hippocampal tissue hypoxia-inducible factor-1α (HIF-1α), and also inhibits the up regulation of MMP-13	[63]
Gouty arthritis(GA)	Hyperuricemia and urate crystal-induced inflammation	Rhein within the physiological levels of humans showed no toxicity on the cell viability and differentiation, but significantly decreased the production of IL-1β, TNF-α and caspase-1 protease in urate crystal-activated macrophages	[26]
		Rhein could inhibit the xanthine oxidase(XOD) activity	[69]
Other arthropathies	Bone resorption in osteoclast	Rhein was validated for its inhibitory effects on the formation of TRAP-positive multinucleated cells and bone resorption	[70]
		Rhein dose-dependently and statistically inhibited osteocalcin release, a situation explained by a reduction of mRNA levels for osteocalcin	[71]

## Discussion and Prospects

Inflammatory responses act as a double-edged sword that protects the body as well as causes indelible damage to it. In specific, inflammatory responses clear pathogens and the damaged cells and initiate the healing process while causing tissue and cellular disorders, inducing autoimmune and other inflammatory diseases. The onset of inflammation activates multiple signaling pathways including NF-kB pathway, MAPK pathway, VEGF pathway and PPARs pathway. Rhein exhibits anti-inflammatory activity through multiple molecular mechanisms, involving the inhibition of signaling pathways and the regulation of cellular processes. Hence, the regulations of rhein on arthritis results from its effects in inhibiting pro-inflammatory cytokine expression, blocking the production and release of inflammatory mediators and inhibiting the onset of inflammatory responses. Moreover, it is worth mentioning that different concentrations of rhein have different effects on signaling pathways, i.e., the efficacy of rhein is dose-dependent. This review summarized the anti-inflammatory effects and mechanisms of rhein and its applications in treating arthritis from a systematic and holistic perspective to provide reference for its basic researches and clinical applications.
